# Which Costs Matter? Costs Included in Economic Evaluation and their Impact on Decision Uncertainty for Stable Coronary Artery Disease

**DOI:** 10.1007/s41669-018-0068-1

**Published:** 2018-02-14

**Authors:** James Lomas, Miqdad Asaria, Laura Bojke, Chris P. Gale, Gerry Richardson, Simon Walker

**Affiliations:** 1 Centre for Health Economics, University of York, York, YO10 5DD UK; 2 MRC Bioinformatics Centre, LICAMM, University of Leeds, Leeds, UK

## Abstract

**Background:**

Variation exists in the resource categories included in economic evaluations, and National Institute for Health and Care Excellence (NICE) guidance suggests the inclusion only of costs related to the index condition or intervention. However, there is a growing consensus that all healthcare costs should be included in economic evaluations for Health Technology Assessments (HTAs), particularly those related to extended years of life.

**Objective and Methods:**

We aimed to quantify the impact of a range of cost categories on the adoption decision about a hypothetical intervention, and uncertainty around that decision, for stable coronary artery disease (SCAD) based on a dataset comprising 94,966 patients. Three costing scenarios were considered: coronary heart disease (CHD) costs only, cardiovascular disease (CVD) costs and all costs. The first two illustrate different interpretations of what might be regarded as related costs.

**Results:**

Employing a 20-year time horizon, the highest mean expected incremental cost was when all costs were included (£2468) and the lowest when CVD costs only were included (£2377). The probability of the treatment being cost effective, estimating health opportunity costs using a ratio of £30,000 per quality-adjusted life-year (QALY), was different for each of the CHD (70%) costs, CVD costs (73%) and all costs (56%) scenarios. The results concern a hypothetical intervention and are illustrative only, as such they cannot necessarily be generalised to all interventions and diseases.

**Conclusions:**

Cost categories included in an economic evaluation of SCAD impact on estimates of both cost effectiveness and decision uncertainty. With an aging and co-morbid population, the inclusion of all healthcare costs may have important ramifications for the selection of healthcare provision on economic grounds.

**Electronic supplementary material:**

The online version of this article (10.1007/s41669-018-0068-1) contains supplementary material, which is available to authorized users.

## Key Points for Decision Makers

**Table:** 

Variation exists in the resource categories included in economic evaluations, and National Institute for Health and Care Excellence (NICE) guidance suggests the inclusion only of costs related to the index condition or intervention.
Cost categories included in an economic evaluation of stable coronary artery disease significantly impact on estimates of cost effectiveness and decision uncertainty.

## Introduction

Interventions in patients with coronary heart disease (CHD) or at risk of CHD present significant costs to the UK National Health Service (NHS), may reduce the risks of CHD events such as acute myocardial infarction (MI), may reduce the risk of other (non-CHD) health events and can potentially improve survival patients [1, 2]. One example is the prescription of statins (HMG-CoA reductase inhibitors) as primary prevention for CHD, which has attracted controversy due to the potential high up-front cost but a potential overall net saving due to reduced future CHD costs as well as wider cardiovascular disease (CVD) costs such as those of strokes avoided [1]. Yet the statins may also increase life expectancy and costs will be incurred by the NHS during these extended years of life [3, 4].

In an economic evaluation the incremental costs incurred by a new intervention and the health benefit it generates are compared to the health benefit of activities that could have been funded with the same resources elsewhere [5]. There is extensive debate, however, around what types of costs should be included in estimating the incremental costs of a new intervention [6, 7], and what costs are included can have material impacts on the expected cost effectiveness and the associated uncertainty. This can affect the decisions reached by policy makers, e.g. whether to approve or reject the new intervention unconditionally, or whether to recommend an alternative coverage decision such as coverage with evidence development [8–10]. This paper considers the importance of the choice of costs to include using a hypothetical intervention that reduces the risk of CVD events in patients with stable coronary artery disease (SCAD).

The debate considers two types of costs: related and unrelated. Garber and Phelps [11] define unrelated costs as those that are independent of the intervention under consideration, and in cases where treatments extend life costs are defined as unrelated if they are independent of treatment but conditional on survival. Conversely, all other costs that are not independent are defined as related. National Institute for Health and Care Excellence (NICE) guidance [12] explicitly makes the recommendation that “costs that are considered to be unrelated to the condition or technology of interest should be excluded”.^1^ A posited theoretical justification for this recommendation is that the aim of economic evaluation is to judge each intervention on its own ‘merits’ [13]. For example, it is argued that the inclusion of future unrelated costs can increase the likelihood of a life-extending technology being judged to not be cost effective even at zero price, which may seem counter-intuitive [7, 14]. The second US Panel on Cost-effectiveness in Health and Medicine, however, recommends that all current and future, related and unrelated healthcare costs should be included in cost-effectiveness analysis. This reflects a growing consensus that all healthcare costs should be included, particularly in extended years of life [3, 15]. Indeed, the guidelines employed in the Netherlands and by the LFN (Dental and Pharmaceutical Benefits Board) in Sweden [16] also recommend the inclusion of future unrelated costs [17].^2^

In practice, whether costs are related or unrelated is based on a judgement of whether they are plausibly related to either the condition or the intervention being considered. This is a subjective assessment and is argued to result in analyses that impose arbitrary restrictions on the costs that are considered relevant [18]. Further, it is not always clear which costs are related and which are unrelated to the condition or intervention. One such example is the case of acute MI and, in particular, how it affects survivors in terms of life expectancy and the likelihood of experiencing health events or diseases in later life (20.72% of patients with SCAD are MI survivors in the CALIBER [ClinicAl research using Linked Bespoke studies and Electronic Records] dataset [19]). MI survivorship is increasing due to the availability of effective treatments; for example, in England in 2010, on average, 27.32% of MI cases that were admitted to hospital died within 30 days, where the corresponding 2002 proportion was 37.18% [20].

A review of published economic evaluations of the treatment of MI finds considerable variation in the types of costs included, reflecting both the subjectivity in determining relevance and variation in recommendations among guidelines. Nineteen economic evaluations published since 2006 are categorised according to the cost categories included: CHD costs only, CVD costs only or all costs (see Electronic Supplementary Material Appendix). CHD costs form the smallest cost category, including costs exclusively related to CHD, e.g. costs attributable to MI. CVD costs include not only all costs attributable to CHD but all costs related to the cardiovascular system more broadly, e.g. ischaemic and haemorrhagic stroke. The CHD costs category is therefore a subset of the CVD costs category. These categories are both subsets of the all costs category, where all costs are included regardless of to which health conditions they are attributable, e.g. they therefore include CVD and non-CVD costs such as those attributable to cancer. Of the 19 studies, five were found to include CHD costs only, eight CVD costs only and six evaluations included all costs. The main difference between studies including CVD costs rather than CHD costs was that costs from stroke events were included. Studies considering related costs only tended to be those that were trial-based analyses, where only CHD/CVD-related data were recorded, or model-based approaches including only CHD or CVD events. One observational data study considered only CVD costs where the analysis was performed on a dataset with detailed information collected on only a select number of CVD endpoints [21]. Of those studies that incorporated non-CVD costs, it is informative to consider the different approaches taken. One study was based on a trial that had collected a wide range of types of resource use [22].^3^ Model-based analyses used a number of different approaches: national averages [23], age-specific costs from risk-adjustment studies [24, 25], estimates of lifetime costs obtained from registry data and use of expert opinion [26]. Finally, for one study undertaking an analysis of observational data, all costs were included in the analysis as the default, but a sensitivity analysis was undertaken in which only costs with the ischaemic heart diseases International Classification of Diseases (ICD) code were included [27].

Given the variation in practice by analysts in conducting economic evaluations of MI treatment and the difficulty in distinguishing what costs are related from those that are not, it is important to consider the implications of which costs are included in an economic evaluation. In this paper a recently published ‘real-world’ cost-effectiveness model is used to consider a hypothetical intervention that reduces the risk of CVD health events for patients with SCAD to investigate the impact of including different cost categories on expected cost effectiveness and decision uncertainty. This analysis represents the first attempt to consider the implications of collecting broader cost data not only in terms of addressing concerns around the expected incremental costs, but also in terms of any impacts upon the associated decision uncertainty.

## Methods

In order to illustrate and quantify the importance, in terms of incremental cost and decision uncertainty, of the analyst’s decision around which cost categories to include, three different scenarios are specified using the CALIBER model [28]. The three scenarios pertain to the range of costs that are included in each analysis: CHD costs only, CVD costs or all costs. Full details of the model are presented elsewhere [28] but, in brief, the CALIBER model employs a set of risk equations estimating probabilities of subsequent MI, ischaemic and haemorrhagic stroke and mortality that are structured as a Markov model to model disease progression in a secondary prevention of CHD context [28]. These equations are estimated using data from four data sources (detailed in Denaxas et al. [29]): the Clinical Practice Research Database (CPRD), Myocardial Ischaemia National Audit Project (MINAP) registry, Hospital Episode Statistics (HES) and Office for National Statistics (ONS).^4^ It is assumed that a hypothetical treatment administered to patients in the SCAD health state at a cost of £250 per year reduces the probability of potentially fatal cardiovascular health events compared with a standard care control group, with a relative risk of 0.8. The patient cohort modelled is assumed to be in the fifth decile of 5-year risk of a composite CVD first event.

The model is evaluated probabilistically, with 999 iterations run in total. For each iteration of the model eight key results are recorded: quality-adjusted life-years (QALYs) associated with the intervention and control groups and costs associated with the intervention and control groups, according to three different costing scenarios. Two of the three scenarios represent plausible approaches to including only related costs as per the NICE guidance on this issue, one of which is narrow and the other slightly broader. In the CHD costs only and CVD costs only scenarios, only healthcare costs that are identifiable as CHD and CVD through the ICD 10th edition (ICD-10) codes of the recorded Health Resource Group (HRG) are included.^5^ The third scenario includes all healthcare costs and so represents an approach where no distinction is drawn in terms of related and unrelated costs. In all scenarios, incremental discounted costs and QALYs are evaluated at a time horizon of 20 years (equivalent to a lifetime time horizon given the starting age of the cohort [mean age at cohort entry was 65 years for males and 73 years for females], where the lifetime time horizon is the recommended choice for treatments that will impact on mortality [5]) using an annual discount rate of 3.5% for both costs and QALYs, and the net health benefit is calculated using £30,000 per QALY [12]. It is not possible within this model to separate out the implications of including different cost categories into effects from unrelated costs in extended years of life and those in years of life not extended by the intervention as the treatment has both morbidity and mortality impacts. However, the relative size of the effect of extended years of life will be smaller with shorter time horizons and so we present some results to show the effect of time horizon on cost effectiveness and decision uncertainty.

## Results

Cost results are first considered in isolation. The mean costs in the treated and control groups are presented along with summary statistics of the incremental costs, illustrating the effect of the three different scenarios on the location and dispersion of the distribution of estimated incremental costs, in Table 1.

**Table 1:** Summary statistics of costs and incremental costs at a time horizon of 20 years

Statistics	Resource categories included		
	CHD (£)	CVD (£)	All (£)
Mean *c*_t_	29,117	36,310	50,202
Mean *c*_c_	26,721	33,934	47,734
Mean *c*_t_ − *c*_c_	2396	2377	2468
Minimum *c*_t_ − *c*_c_	2287	2232	2273
Maximum *c*_t_ − *c*_c_	2523	2547	2686
Range *c*_t_ − *c*_c_	237	315	414
Standard deviation *c*_t_ − *c*_c_	39	53	69
Median *c*_t_ − *c*_c_	2394	2374	2468
Q_0.025_ *c*_t_ − *c*_c_	2323	2281	2343
Q_0.975_ *c*_t_ − *c*_c_	2471	2478	2595
95% credibility interval *c*_t_ − *c*_c_	148	197	252

*CHD* coronary artery disease, *CVD* cardiovascular disease, *c*_*c*_ cost when control, *c*_*t*_ cost when treated, *Q*_*0.025*_ 2.5th percentile, *Q*_*0.975*_ 97.5th percentile

**Fig. 1:**
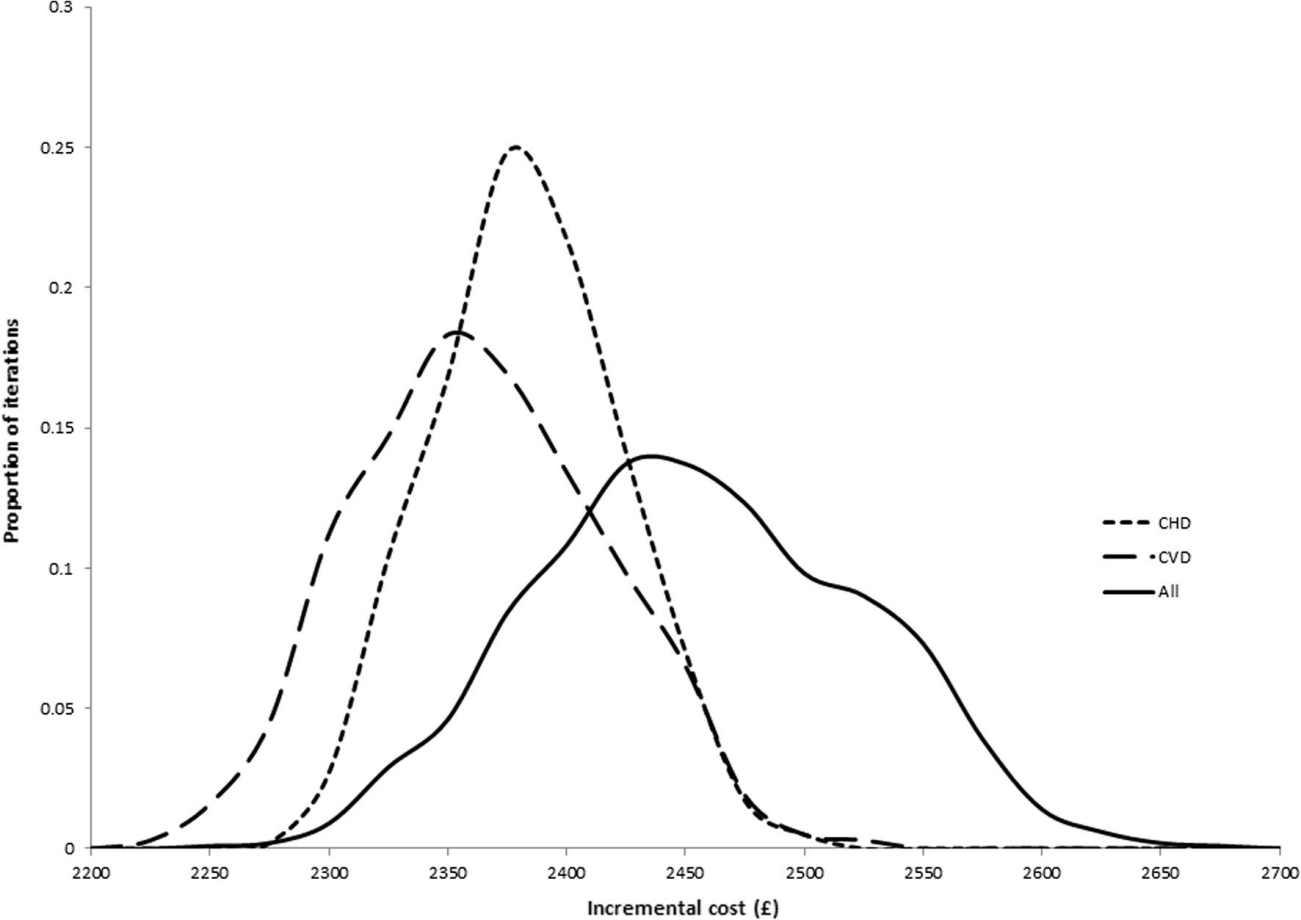
Frequency plot of incremental costs under three scenarios. *CHD* coronary heart disease, *CVD* cardiovascular disease

Mean costs over a 20-year time horizon for both the treated and control groups increase with the inclusion of additional cost categories. In terms of incremental costs, including all costs results in the highest mean incremental cost. The CVD costs only scenario gives the lowest mean incremental cost. Variability of incremental costs increases when more cost categories are included based on the width of the 95% credibility interval. The distribution of incremental costs is presented in Fig. 1.

Figure 1 shows the distributions of incremental costs under each scenario. Mean incremental costs were lowest in the CVD costs only scenario and highest in the all costs scenario. The distributions of incremental costs under scenarios with more cost categories included are considerably wider, reflecting greater variability.

**Fig. 2:**
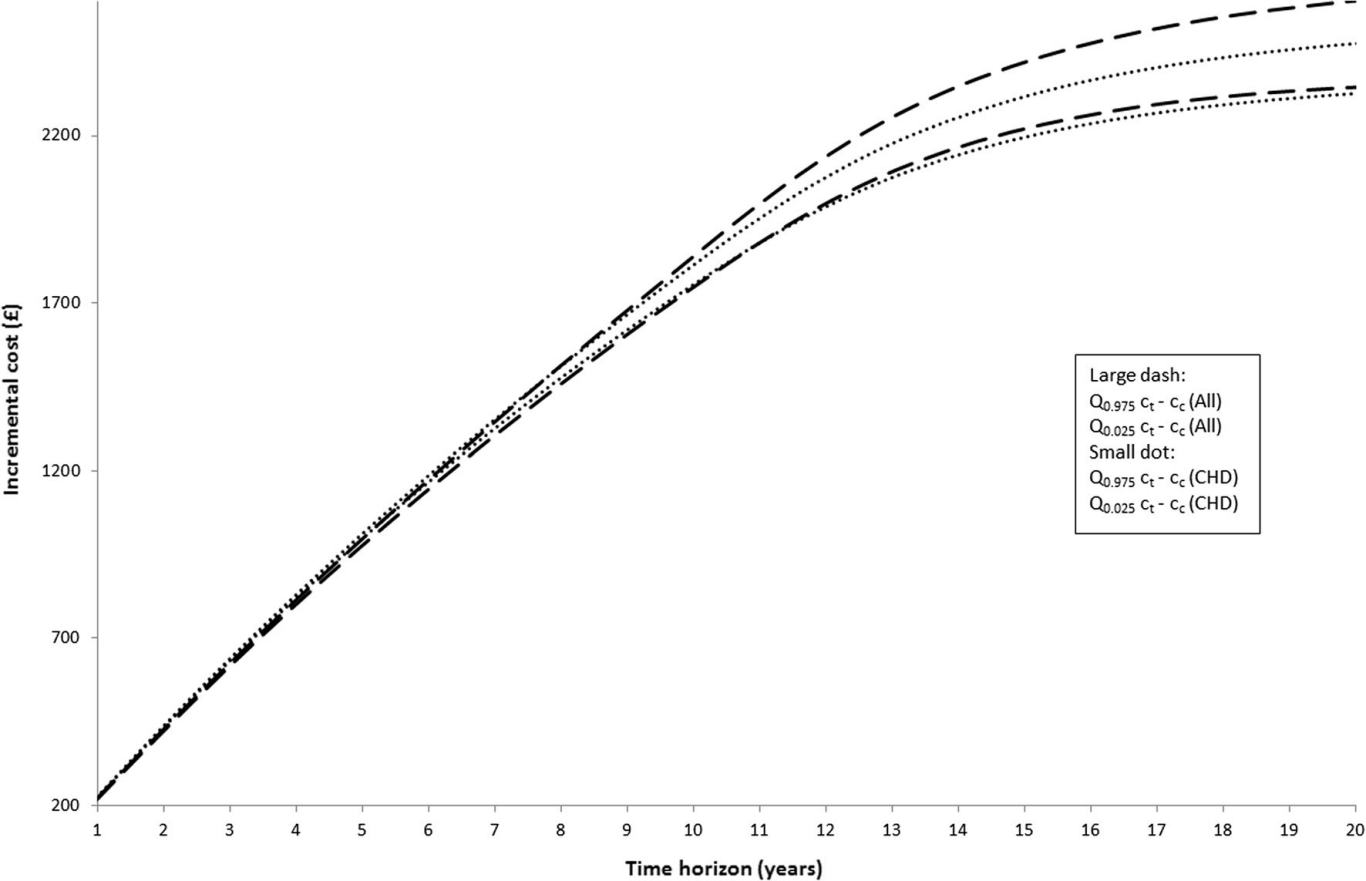
95% credibility interval (CI) against time horizon by costing scenario. *CHD* coronary artery disease, *c*_*c*_ cost when control, *c*_*t*_ cost when treated, *Q*_*0.025*_ 2.5th percentile, *Q*_*0.975*_ 97.5th percentile

**Fig. 3:**
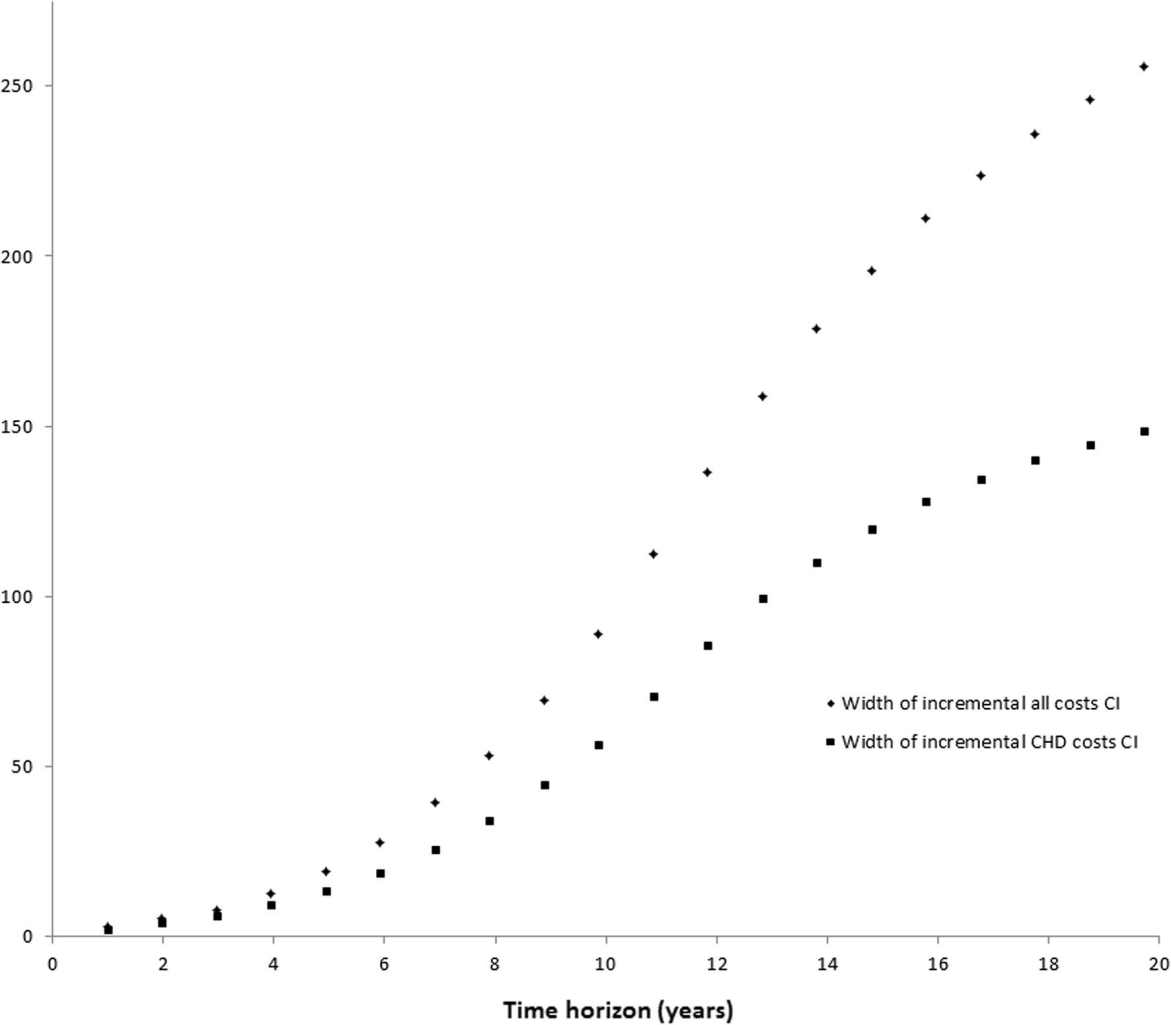
Width of 95% credibility interval against time horizon by costing scenario. *CHD* coronary heart disease, *CI* 95% credibility interval

To investigate the relative effect of unrelated costs in extended years of life compared with those in life-years not extended by the intervention, we investigate the distribution of incremental costs for different time horizons up to 20 years, with changes over time likely to reflect the increasing impact of costs in extended years of life. These results are summarised in Figs. 2 and 3.

Figure 2 shows the credibility interval of the incremental costs for two different cost category scenarios (CHD and all costs) against the time horizon. It can be seen that with longer time horizons, such as 20 years, the all costs scenario produces higher incremental costs on average. Looking at the shorter time horizons the opposite is found, with incremental costs on average higher under the CHD scenario. Variability, seen from the width of the credibility interval shown in Fig. 3, is higher for the all costs scenario at all time horizons. For both scenarios, variability increases with the time horizon.

It is also useful to consider the impact of incremental costs on cost effectiveness in order to illustrate how these findings could affect decision-making and decision uncertainty. In all scenarios the mean incremental QALY from treatment is 0.084. These are the denominators in the estimates of the incremental cost-effectiveness ratios (ICERs) provided in Table 2, where the numerators are the mean incremental costs reported in Table 1.

**Table 2:** Estimated incremental cost-effectiveness ratios in the different costing scenarios

	Resource categories included		
	CHD	CVD	All
ICER	£28,626 per QALY	£28,395 per QALY	£29,485 per QALY

*CHD* coronary heart disease, *CVD* cardiovascular disease, *ICER* incremental cost-effectiveness ratio, *QALY* quality-adjusted life-year

**Fig. 4:**
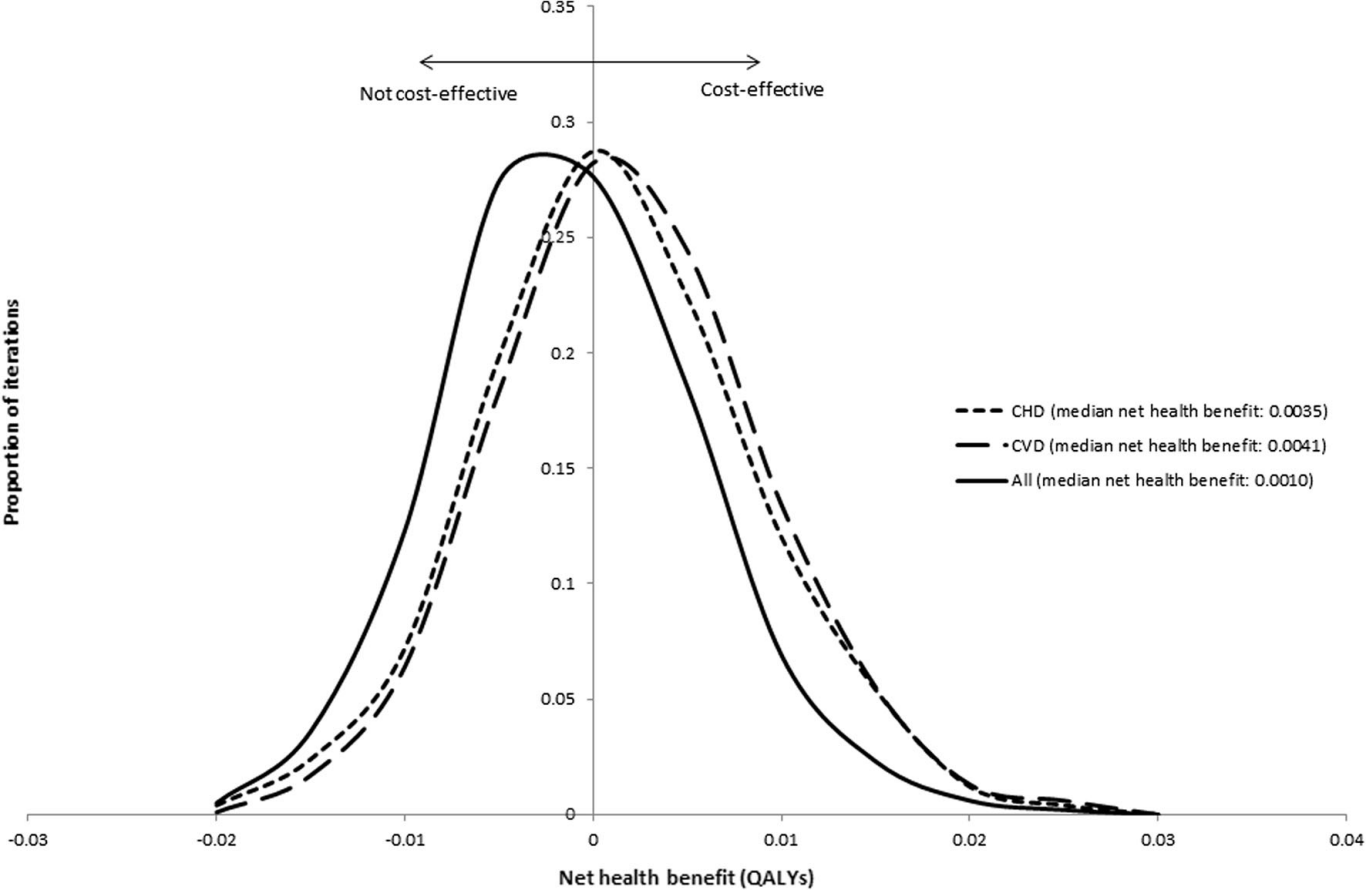
Frequency plot of net health benefits under three scenarios. *CHD* coronary heart disease, *CVD* cardiovascular disease, *QALYs* quality-adjusted life-years

For illustrative purposes we consider a decision maker who regards a treatment as cost effective when the ICER is less than £30,000 per QALY gained [12]. Whilst the treatment is expected to be cost effective in all scenarios (all of the ICERs are below £30,000 per QALY), it can be seen that the choice of cost categories that are included is likely to influence the degree of decision uncertainty. Figure 4 shows the distributions of net health benefits for the treatment. The treatment is cost effective when the net health benefit is greater than zero.

The proportion of iterations with a positive net health benefit, i.e. the probability of being cost effective, varies across the three scenarios: CHD costs (70%), CVD costs (73%) and all costs (56%). These percentages reflect the distribution of net health benefit in the different scenarios where both the location and dispersion of the distributions is influenced by the choice of cost categories to be included.

## Discussion

This paper assesses the cost effectiveness of a hypothetical intervention using a previously published model based on ‘real-world’ data to demonstrate that the inclusion of unrelated costs will affect both the mean incremental costs, ICER and decision uncertainty.

Unambiguously, costs will be greater when more categories are included (given costs are non-negative). It is harder to predict how the different cost category scenarios will affect the estimated incremental costs that are required for economic evaluation. The treatment under consideration here reduces the risk of cardiovascular events, each with associated CHD, CVD and non-CVD costs, in normal years of life (i.e. not extended years of life). Each of these events is potentially fatal, and so the treatment extends life. This distinction is important when thinking about the overall effect on incremental costs. In normal years of life, including more cost categories increases the magnitude of the cost savings that are brought about by the reduced risk of cardiovascular events.^6^ However, including additional cost categories means greater costs in extended years of life resulting from the treatment. As such, these two effects offset one another to some extent.^7^ Therefore, the overall effect is ambiguous. Moving from only CHD costs to CVD costs reduces the mean incremental cost, because more cost savings (e.g. from reduced strokes) are captured during normal years of life than the additional incremental CVD costs captured in extended years of life resulting from the treatment. However, moving from either CHD costs only or CVD costs only to including all costs increases the incremental costs overall because the extended years of life effect outweighs the effect of capturing more savings during normal years of life. Unfortunately, given the model used, it is not possible to separate out these effects and only the joint effect is observable. However, by considering shorter time horizons we can examine the relative impacts of costs in normal and extended years of life. With a shorter time horizon the effect of costs in normal years of life is relatively more influential and so the direction of effect on the cost effectiveness of the ambiguous relationship between inclusion of broader cost categories may vary with the time horizon. Indeed, this is what is seen in our results. At very short time horizons, the incremental costs are on average higher when restricted to CHD costs only than when including all cost categories. The variability of costs is higher for all costs than it is for CHD costs at all time horizons analysed. This would translate to greater decision uncertainty when including all costs, ceteris paribus, even when using short time horizons.

In addition to the effect on mean incremental costs, this paper also explores the effect that the different scenarios have on decision uncertainty. Again, this effect needs to be thought of as two separate underlying processes. The first of these is, when considering a technology that has positive incremental costs and incremental QALYs, the proximity of the mean ICER to the ‘cost-effectiveness threshold’ being used in the analysis.^8^ When the ICER is close to the ‘cost-effectiveness threshold’, ceteris paribus, then it is more likely that the decision is going to be uncertain. The other factor is the uncertainty of the estimated ICER (in this case driven by the variability in incremental costs). The variability of costs (and incremental costs) is likely to increase with the addition of cost categories, unless a negative correlation exists between the costs within the narrower category and those only within the broader category.

Where there is no extension of life effect from a treatment, including unrelated costs will not affect the mean ICER, in expectation, as these additional costs would be the same on average regardless of treatment received. Decision uncertainty, however, will be affected given the variability of costs. As such, under these circumstances, the addition of truly unrelated costs amounts to adding noise and increased variability into the model.

Adding noise and variability can lead to greater uncertainty and there are two reasons why this might be a concern. The first is that there will be reduced power to detect a statistically significant effect, since standard errors will increase. The second concern, more prevalent within health economic evaluation, is that the decision uncertainty will be inflated, thus reducing the probability of making the right decision. Of course, the analyst will never know what is truly related and truly unrelated and so identifying something as noise with certainty is impossible. This is not to say that unrelated costs should never be included because of the potential of introducing noise, but it is important to consider the implications of adding in what might be truly unrelated costs in normal years of life. This issue has been overlooked in the literature on this topic to date.

As already discussed, there is reason to caution against including unrelated costs in some cases where there is no (or little) mortality effect. In addition, obtaining data on unrelated costs may not be without cost to the analyst, especially obtaining data that are both very unlikely to be related and are realised many years in the future—although this may be getting less costly with time [4] as a growing number of sources exist that can be used to provide data for unrelated costs in extended years of life across a number of different countries [30–34].

Lack of appropriate and robust data will be an issue in many circumstances. Experimental evidence typically does not have a sufficiently long time horizon to identify the occurrence of all future related and unrelated events and their associated costs. As an alternative, the use of observational data from administrative sources is becoming increasingly prevalent and is well-suited to estimate parameters with large numbers of observations. Observational data are typically collected over a longer period of time; however, by design it is not as robust as randomised trial data when it comes to estimating the incremental cost caused by treatment, with potential bias coming from unobserved confounding and selection effects. With both randomised controlled trial (RCT) and observational evidence there is the issue of sufficient breadth of study design to identify all related and unrelated costs. By their very nature, such studies would be unlikely to be focused on collecting events unrelated to the primary intervention and, as such, data collection would have to be sufficiently flexible or broad to capture all costs (related and unrelated). Routine data sources, such as HES in the UK context, which capture all healthcare-related interventions and hospital visits are another possibility. HES has recently been used to compute average costs by age, sex and index of multiple deprivation (IMD) group [34]. This source has the potential to be very useful for incorporation into decision models, but there are two limitations that provide scope for future research. The first is that the costs in this paper include both related and unrelated costs, meaning that researchers would need to adapt its results in order to include it alongside related costs in extended years of life estimated as part of the model [33]. The second limitation is that the costs of the patient population at hand may not be well represented by the national average on account of the relationship between costs and co-morbidities. In such circumstances, disentangling the associations between costs, age and morbidities will be required for the precise estimation of parameters capturing unrelated costs. There is already a growing understanding of the causal effect that age has on costs, which has been found to be questionable. In the first instance, age was considered to be a ‘red herring’, with costs increasing with age due only to a spurious association, and the underlying effect on costs deriving from time until death and not age itself [35, 36]. More recently, research has started to question whether this red herring hypothesis is itself a red herring, with researchers analysing the effects of morbidity and multi-morbidity on costs in greater depth [37, 38]. Despite these limitations and challenges it is likely that, with further research, electronic health records will be increasingly utilised for the incorporation of unrelated costs in extended years of life where this is deemed appropriate.

Even in the absence of observational data, it is still possible to place a reasonable estimate on these future unrelated costs parameters. There are methods that can be used to illustrate the potential range values for these parameters, reflecting their degree of uncertainty [39]. This can then be used to guide future research priorities [40]. To inform these uncertain estimates, expert elicitation methods could be useful. In some circumstances, even where long-term observational data are not available, clinical experts may have experience of observing events occurring in survivors of acute MI over a long enough period. This clinical experience can be used to generate distributions for these unrelated future costs, reflecting a large amount of uncertainty around the estimates. All things considered, lack of data is certainly a challenge, but this seems more of a practical obstacle to overcome (if these data are potentially influential to the decision) rather than a reason for exclusion.

### Limitations

Because the model itself concerns a hypothetical intervention, the specific results should not be over-interpreted, nor can they be generalised for assessments of how much of an impact the inclusion of unrelated costs will have in different contexts. The results do allow us to identify the types of effects that will result from including unrelated costs. To put this another way, this paper contributes to the literature by illustrating *which* costs matter and *why*, but should not be used to say *how much* different costs matter outside of the context analysed. For example, within the context analysed, it can be seen that the variability of incremental costs is generally low, irrespective of which costing scenario is used. This results from the large number of observations upon which the system of equations is estimated, and hence granting a degree of precision that would not be attainable in smaller studies.

## Conclusions

It is suggested that economic evaluations should include estimates of incremental healthcare costs that fall within the healthcare budget and not just the intervention costs associated with the treatment. However, in moving beyond intervention costs, the analyst faces challenges in determining which healthcare costs (unrelated and unrelated) are truly caused by the treatment, which can be particularly demanding when there are variable and noisy cost data, and when the knowledge around the natural history of a disease is not well-known, as in the case of acute MI survivorship. With an aging and co-morbid population, the inclusion of all healthcare costs may have important ramifications for the selection of healthcare provision on economic grounds.

This paper contributes to this discussion by illustrating the importance of different types of costs—*which costs matter*—in the context of SCAD, where little is known about what is truly related or unrelated and there is considerable variation in analytical practice concerning cost categories to include in economic evaluation. It has been shown that costs matter, not just in terms of the point estimate of cost effectiveness, the expected ICER, but also in terms of decision uncertainty. Where adding unrelated costs in evaluations without an impact on survival, this may simply add noise to the data and therefore will reduce the probability of making the right decision.

The inclusion of unrelated costs is important, especially when the treatment is expected to extend life, and so including all cost categories is at least recommended as a sensitivity analysis (if the associated cost to analyst of obtaining data is not prohibitively high).

## Electronic supplementary material

Below is the link to the electronic supplementary material.

Supplementary material 1 (DOC 85 kb)

## Footnotes

1 A fuller discussion of guidelines and recommendations pertaining to the exclusion of unrelated costs (in particular those in extended years of life) can be found elsewhere [41].

2 A further debate surrounds the inclusion of non-healthcare costs, as recommended by the guidelines of the Netherlands and Sweden. This remains more controversial and is beyond the scope of this paper.

3 Interestingly, in this study one of the resource use measures that was particularly high for one patient among those treated was considered by the authors to only be “weakly related” to the intervention and so was excluded as part of the sensitivity analysis, which had quite a large effect on the mean cost difference [22].

4 Further details given in Asaria et al. [28] and Walker et al. [42]. Resource use is taken from administrative data sources, CPRD and HES, and then costed on the basis of NHS reference costs, Personal Social Services Research Unit (PSSRU) unit costs and NHS prescription cost analysis [43–45]. Estimation of cost parameters in the model is undertaken using the panel data approach outlined in Walker et al. [42].

5 CHD HRGs were defined as those with ICD-10 codes from I10 through to I52.

6 Note that this holds only if the excluded cost categories are not truly unrelated.

7 Authors have noted this kind of offset in previous applications [46].

8 In other words, the proximity of the mean net health benefit of the technology to zero.
