# Supplementary material for: Which Costs Matter? Costs Included in Economic Evaluation and their Impact on Decision Uncertainty for Stable Coronary Artery Disease
Source: Pharmacoecon Open. 2018 Feb 14;2(4):403–13. doi: 10.1007/s41669-018-0068-1 (PMC6249199; doi:10.1007/s41669-018-0068-1)
Supplement: Supplementary file 1 — Supplementary material 1 (DOC 85 kb) [file 41669_2018_68_MOESM1_ESM.doc]

## Appendix

NHS EED was searched using the following search strategy:

((myocardial infarction):TI) and ((Economic evaluation:ZDT and Abstract:ZPS)) FROM 2006 TO 2016

Twenty records were identified of which one was considered to not be a cost-effectiveness analysis, the following nineteen publications were analysed.

| DOI: 10.1016/j.jval.2012.01.004 |
| --- |
| TTL: Cost-effectiveness of aldosterone antagonists for the treatment of post-myocardial infarction heart failure |
| AUT: McKenna C, Walker S, Lorgelly P, Fenwick E, Burch J, Suekarran S, Bakhai A, Witte K, Harden M, Wright K, Woolacott N, Palmer S |
| XSO: Value in Health |
| XYR: 2012 |
| VOL: 15(3) |
| PAG: 420-428 |
|  |
| DOI: 10.1136/heartjnl-2011-301323 |
| TTL: Cost-effectiveness of bivalirudin versus heparin plus glycoprotein IIb/IIIa inhibitor in the treatment of acute ST-segment elevation myocardial infarction |
| AUT: Schwenkglenks M, Toward TJ, Plent S, Szucs TD, Blackman DJ, Baumbach A |
| XSO: Heart |
| XYR: 2012 |
| VOL: 98(7) |
| PAG: 544-551 |
|  |
| DOI: 10.2165/11597340-000000000-00000 |
| TTL: Cost effectiveness of drug-eluting stents in acute myocardial infarction patients in Germany: results from administrative data using a propensity score-matching approach |
| AUT: Baumler M, Stargardt T, Schreyogg J, Busse R |
| XSO: Applied Health Economics and Health Policy |
| XYR: 2012 |
| VOL: 10(4) |
| PAG: 235-248 |
|  |
| DOI: 10.1136/heartjnl-2012-302188 |
| TTL: Cost-effectiveness of presentation versus delayed troponin testing for acute myocardial infarction |
| AUT: Thokala P, Goodacre SW, Collinson PO, Stevens JW, Mills NL, Newby DE, Morris F, Kendall J, Stevenson MD |
| XSO: Heart |
| XYR: 2012 |
| VOL: 98(20) |
| PAG: 1498-1503 |
|  |
| TTL: Cost-effectiveness analysis of percutaneous coronary intervention versus thrombolytic therapy in patients with an ST-elevated myocardial infarction |
| AUT: Iva G |
| XSO: Serbian Journal of Experimental and Clinical Research |
| XYR: 2011 |
| VOL: 12(4) |
| PAG: 147-152 |
|  |
| DOI: 10.1016/j.jval.2011.02.1180 |
| TTL: Cost-effectiveness of implantable defibrillators after myocardial infarction based on 8-year follow-up data (MADIT II) |
| AUT: Gandjour A, Holler A, Adarkwah CC |
| XSO: Value in Health |
| XYR: 2011 |
| VOL: 14(6) |
| PAG: 812-817 |
|  |
| DOI: 10.1111/j.1553-2712.2011.01068.x |
| TTL: Cost-effectiveness of point-of-care biomarker assessment for suspected myocardial infarction: the Randomized Assessment of Treatment using Panel Assay of Cardiac markers (RATPAC) trial |
| AUT: Fitzgerald P, Goodacre SW, Cross E, Dixon S, Randomized Assessment of Treatment using Panel Assay of Cardiac markers Research Team |
| XSO: Academic Emergency Medicine |
| XYR: 2011 |
| VOL: 18(5) |
| PAG: 488-495 |
|  |
| DOI: 10.1001/archinternmed.2010.479 |
| TTL: Projected cost-effectiveness of smoking cessation interventions in patients hospitalized with myocardial infarction |
| AUT: Ladapo JA, Jaffer FA, Weinstein MC, Froelicher ES |
| XSO: Archives of Internal Medicine |
| XYR: 2011 |
| VOL: 171(1) |
| PAG: 39-45 |
|  |
| DOI: 10.1161/CIRCOUTCOMES.109.908541 |
| TTL: Comparative effectiveness of ST-segment-elevation myocardial infarction regionalization strategies |
| AUT: Concannon TW, Kent DM, Normand SL, Newhouse JP, Griffith JL, Cohen J, Beshansky JR, Wong JB, Aversano T, Selker HP |
| XSO: Circulation: Cardiovascular Quality and Outcomes |
| XYR: 2010 |
| VOL: 3(5) |
| PAG: 506-513 |
|  |
| DOI: 10.1161/CIRCULATIONAHA.109.900704 |
| TTL: Cost-effectiveness of prasugrel versus clopidogrel in patients with acute coronary syndromes and planned percutaneous coronary intervention: results from the Trial to Assess Improvement in Therapeutic Outcomes by Optimizing Platelet Inhibition With Prasugrel - Thrombolysis in Myocardial Infarction TRITON-TIMI 38 |
| AUT: Mahoney EM, Wang K, Arnold SV, Proskorovsky I, Wiviott S, Antman E, Braunwald E, Cohen DJ |
| XSO: Circulation |
| XYR: 2010 |
| VOL: 121(1) |
| PAG: 71-79 |
|  |
| DOI: 10.1007/s12325-010-0013-x |
| TTL: Enoxaparin is a cost-effective adjunct to fibrinolytic therapy for ST-elevation myocardial infarction in contemporary practice |
| AUT: Menown I, Montalescot G, Pal N, Fidler C, Orme M, Gillard S |
| XSO: Advances in Therapy |
| XYR: 2010 |
| VOL: 27(3) |
| PAG: 181-191 |
|  |
| DOI: 10.1136/hrt.2009.167130 |
| TTL: Primary angioplasty versus thrombolysis for acute ST-elevation myocardial infarction: an economic analysis of the National Infarct Angioplasty Project |
| AUT: Wailoo A, Goodacre S, Sampson F, Hernandez Alava M, Asseburg C, Palmer S, Sculpher M, Abrams K, de Belder M, Gray H |
| XSO: Heart |
| XYR: 2010 |
| VOL: 96(9) |
| PAG: 668-672 |
|  |
| TTL: Cost-effectiveness of enoxaparin compared with unfractionated heparin in ST elevation myocardial infarction patients undergoing pharmacological reperfusion: a Canadian analysis of the Enoxaparin and Thrombolysis Reperfusion for Acute Myocardial Infarction Treatment - Thrombolysis in Myocardial Infarction (ExTRACT-TIMI) 25 trial |
| AUT: Welsh RC, Sauriol L, Zhang Z, Kolm P, Weintraub WS, Theroux P |
| XSO: Canadian Journal of Cardiology |
| XYR: 2009 |
| VOL: 25(12) |
| PAG: e399-e405 |
|  |
| TTL: Cost-effectiveness of prehospital versus inhospital thrombolysis in acute myocardial infarction |
| AUT: Araujo D V, Tura B R, Brasileiro A L, Neto H L, Pavao A L, Teich V |
| XSO: Arquivos Brasileiros de Cardiologia |
| XYR: 2008 |
| VOL: 90(2) |
| PAG: 91-98 |
|  |
| DOI: 10.1161/CIRCULATIONAHA.107.735605 |
| TTL: Cost-effectiveness of providing full drug coverage to increase medication adherence in post-myocardial infarction Medicare beneficiaries |
| AUT: Choudhry N K, Patrick A R, Antman E M, Avorn J, Shrank W H |
| XSO: Circulation |
| XYR: 2008 |
| VOL: 117 |
| PAG: 1261-1268 |
|  |
| DOI: 10.1016/j.clinthera.2007.06.020 |
| TTL: Cost-effectiveness of clopidogrel in myocardial infarction with ST-segment elevation: a European model based on the CLARITY and COMMIT trials |
| AUT: Berg H, Lindgren P, Spiesser J, Parry D, Jonsson B |
| XSO: Clinical Therapeutics |
| XYR: 2007 |
| VOL: 29(6) |
| PAG: 1184-1202 |
|  |
| DOI: 10.1177/0272989X07306111 |
| TTL: The value of myocardial perfusion scintigraphy in the diagnosis and management of angina and myocardial infarction: a probabilistic economic analysis |
| AUT: Hernandez R, Vale L |
| XSO: Medical Decision Making |
| XYR: 2007 |
| VOL: 27 |
| PAG: 772-788 |
|  |
| TTL: A Mediterranean diet is cost-effective in patients with previous myocardial infarction |
| AUT: Dalziel K, Segal L, De Lorgeril M |
| XSO: Journal of Nutrition |
| XYR: 2006 |
| VOL: 136(7) |
| PAG: 1879-1885 |
|  |
| TTL: Using simulation to estimate the cost effectiveness of improving ambulance and thrombolysis response times after myocardial infarction |
| AUT: Chase D, Roderick P, Cooper K, Davies R, Quinn T, Raftery J |
| XSO: Emergency Medicine Journal |
| XYR: 2006 |
| VOL: 23(1) |
| PAG: 67-72 |
